# Family as a Bridge to Improve Meaning in Latinx Individuals Coping with Cancer

**DOI:** 10.1089/pmr.2022.0035

**Published:** 2022-09-05

**Authors:** Normarie Torres-Blasco, Rosario Costas-Muñiz, Carolina Zamore, Laura Porter, Maria Claros, Guillermo Bernal, Megan J. Shen, William Breitbart, Lianel Rosario, Cristina Peña-Vargas, Eida M. Castro-Figueroa

**Affiliations:** ^1^ Department of Psychiatry and Human Behavior, School of Behavioral and Brain Science, Ponce Research Institute, Ponce Health Sciences University, Ponce, Puerto Rico.; ^2^ Department of Psychiatry and Behavioral Sciences, Memorial Sloan-Kettering Cancer Center, Immigrant Health & Cancer Disparities, New York, New York, USA.; ^3^ Department of Psychiatry and Behavioral Sciences, Duke University Medical Center, Durham, North Carolina, USA.; ^4^ Caribbean Alliance of National Psychological Associations, San Juan, Puerto Rico.; ^5^ Division of Geriatrics and Palliative Medicine, Department of Medicine, New York, New York, USA.

**Keywords:** cancer, family, Latinx

## Abstract

**Background:**

Family and meaning-making resources are culturally congruent and help support Latinx coping with cancer.

**Objectives:**

To explore Latinx advanced cancer patients' perspectives on the role of family and meaning/purpose in adjustment to advanced cancer.

**Methods:**

A qualitative study was conducted. In-depth interviews were performed and transcripts were analyzed using the method from applied thematic analysis.

**Setting/Subject:**

Participants were patients with any advanced cancer diagnosis, recruited from Memorial Sloan Kettering Cancer Center (MSKCC), New York; Lincoln Medical Center (LMC), New York; and Ponce Health Science University (PHSU), Puerto Rico.

**Measurements:**

Sociodemographic and semistructured interview.

**Results:**

*N* = 24 in-depth interviews were completed by Latinx advanced cancer patients (Stage III and IV). When evaluating patients' perspectives on the role of family and meaning/purpose in adjustment to advanced cancer, the team generated three categories: (1) family support, (2) family communication, and (3) include support for the family. Many patients reported the importance of family as a source of meaning and social support in the process of cancer diagnosis and treatment. They also reported communication as central to process information and planning. Also, participants describe their desire to incorporate family members into therapy and for encouraging them to seek counseling.

**Conclusions:**

Results highlight the need to include syntonic cultural values such as family and meaning for Latinx individuals coping with advanced cancer in psychological interventions.

## Introduction

Approximately 38% of Latinx individuals will develop cancer in their lifetime,^1^ and compared with non-Hispanic Whites, Latinx individuals are more likely to be diagnosed at an advanced stage.^2^ Compounding this problem, Latinx individuals are less likely to have adequate access to culturally congruent psychosocial interventions.^3–14^ The lack of interventions designed to be culturally congruent and relevant for Latinx communities contributes to less access to care for under-represented or vulnerable populations in need of culturally appropriate interventions.

Some end-of-life studies conducted with advanced cancer Latinx patients have noted the importance of including caregivers in the patient's treatment decision and the importance of including the caregivers in this process of care.^15^ Additional studies with Latinx cancer patients underscore how family support is essential while coping with cancer^16^ and the importance of family involvement in care.^16–18^ Family support helps meet patients' practical, physical, and emotional needs,^16^ and assists in treatment decision making, advanced care planning, and end-of-life decisions.^19–21^

Moreover, family and meaning-making are resources that may help support Latinx individuals coping with cancer,^16,22^ especially when Latinx patients perceive meaning-making as a coping mechanism,^16^ even when they migrate to other countries.^23–29^

Many factors lead to cancer patients' meaning making, yet the implication of Latinx family-related content is absent in the literature and is also a gap in the development of culturally appropriate interventions. Specifically, when family relationships are a cultural value that may influence the Latinx meaning-making process in advanced cancer and can aid in the development of future interventions. The aim of this study was to explore Latinx advanced cancer patients' perspectives on the role of family and meaning/purpose in adjustment to advanced cancer.

## Materials and Methods

### Participants and procedures

Research staff identified and approached participants (*n* = 127) between August 2015 and October 2018 to adapt a psychotherapy intervention with advanced cancer patients at two cancer clinics in New York and one in Ponce, Puerto Rico. Eligible participants were adult cancer patients (ages 18 years or older) diagnosed with stage III or IV solid tumor cancer, self-identified Latinx/Hispanic ethnicity, fluent in Spanish, and selected by convenience. Of the 127 patients approached, 49% refused to participate, and 9% became ineligible after providing consent (i.e., became too ill to participate), yielding a sample of 54 patients.

A nested sample of the first consecutive 24 patients was invited to complete in-depth and semistructured interviews until saturation was reached. This secondary data analysis aimed to explore Latinx advanced cancer patients' perspectives on the role of family and meaning/purpose in adjustment to advanced cancer. This research was reviewed and approved by the three institutions' review boards/privacy boards.

### Measures

#### Semistructured interviews

Interviews ran ∼90–120 minutes and were conducted in Spanish. The in-depth interview consisted of open-ended questions about the patients' meaning-making processes and coping upon diagnosis, sources of meaning in their lives, spirituality, and meaning making after their cancer diagnosis.

### Analysis

The analyses, integration, and interpretation of the semistructured interview were all completed in Spanish. Initially, 25% of the interviews were coded using an open coding approach; transcribed interviews were coded by marking passages of text with phrases indicating the content of the discussions.^30–32^ Using ATLAS,it's report and query functions, the qualitative analysts (N.T.-B., M.C., C.Z., and R.C.-M.) independently coded the transcripts and discussed divergence and convergence points.^30–32^

These discussions continued until the group reached a consensus on the code's application.^30–32^ A coding dictionary was developed based on the consensus meeting discussions.^30–32^ The qualitative coders then coded the remaining transcriptions using the coding dictionary. Through consensus meetings, divergence and convergence points were discussed among the group until consensus was met. Intercoder reliability was conducted through team-based consensus.

## Results

### Participants

Table 1 summarizes the general characteristics of the patients in this study.

**Table 1.** Participant Demographic Information

Characteristics	***N*** (%)
Age, years	
Mean (SD)	54.17 (13.7)
Gender	
Male	16 (66.7)
Female	8 (33.3)
Marital status	
Married or partnered	20 (83.3)
Single	3 (12.5)
Divorced	1 (4.2)
Education	
Less than high school	6 (25.0)
12th grade/high school graduate	6 (25.0)
Some college or associate degree	8 (33.3)
College graduate	2 (8.3)
Post college/graduate school	2 (8.3)
Employment status	
Employed	12 (50.0)
Retired	6 (25.0)
Unemployed or disabled	6 (25.0)
Race	
White or Caucasian	11 (45.8)
Black	1 (4.2)
Other	12 (50.0)
Dominant language	
English	
Spanish	21 (87.5)
Both	3 (12.5)
Birthplace	
United States	2 (8.3)
Puerto Rico	10 (41.7)
Dominican Republic	3 (12.5)
Mexico	2 (8.3)
Other countries in Latin America	7 (29.2)
Diagnosis	
Breast	6 (25.0)
Prostate	5 (20.8)
Gastrointestinal	3 (12.5)
Other	10 (41.7)
Cancer stage	
III	11 (45.8)
IV	13 (54.2)

Values are no. (%), unless otherwise noted; Percentages may not equal 100%, due to rounding.

SD, standard deviation.

### Perspective of the family of the Latinx cancer patient

Participant narratives were grouped into three broad categories: family support, family communication, and inclusion of family into psychotherapy, see Table 2.

**Table 2.** Categories of Family-Related Content

Category	Theme	Quotes
1. Family as a source of meaning	Source of meaning making	[Referring to sources of meaning] 1. “Sí. Mi esposo, mi familia, mis hermanas. Mis hijas obviamente son mi mayor fuente.” P02 1. “Yes. My husband, my family, my sisters. My daughters are obviously my biggest source.” P02 2. “Yeah, mi hija y mi esposo.” P06 2. “Yeah, my daughter and my husband.” P06 3. “Yo creo que lo único que me ha dado sentido a mi vida así es Dios y mis hijos, para seguir luchando.” P10 3. “I believe that the only thing that has given my life meaning like this is God and my children, to continue fighting.” P10 4. “La fuente de sentido es tratar de seguir adelante junto a mi familia, tratar de vivir mejor más de lo que vivo ahora y ayudar así a las personas…” P12 4. “The source of meaning is trying to move forward with my family, trying to live better than I live now and thus help people.” P12 5. “Mi familia. Mi familia, mi alrededor, lo que hago, eso todo es…” P14 5. “My family. My family, my surroundings, what I do, that's all.” P14 6. “Mi hijo.” P17 6. “My son.” P17
	Source of meaning: humor	[referring to humor] 7. “Cuando yo los veo felices a ellos. Yo ver feliz a mis hijos para mi es todo. No hay cosa más grande que verlos… felices.” P01 7. “When I see them happy. Seeing my children happy is everything to me. There is nothing greater than seeing them… happy.” P01 8. “Sí, cuando veo a mis hijos (Se ríe).” P10 8. “Yes, when I see my children (Laughs).” P10 9. “Ahora veo a otros niños y lo primero que viene son mis niñas, que quiero que así sean de felices, que gocen y ya a uno como que todo le va dando algo más de sentido, de importancia.” P15 9. “Now I see other children and the first thing that comes [to mind] are my girls, who I want them to be happy, to enjoy themselves and it's as if everything is given some more sense of meaning, of importance.” P15 10. “Me reúno con la familia, todo el mundo espera un chiste; siempre algo nuevo.” P20 4. “I meet with the family; everyone expects a joke; always something new.” P20 11. “Tengo, si a veces mi esposa dice estás loco, si mira como estas y no dejas de decir bromas, dice, y digo pues es que reír es bueno, déjame. Y además no me estoy muriendo, estoy de pie, caminando, y a ella le da risa.” P29 11. “I have [referring to having humor in their life], sometimes my wife says ‘you're crazy, look at how you are, and you don't stop making jokes, and I say ‘because laughing is good, let me [keep joking]. And besides, I'm not dying, I'm standing, walking, and she laughs.” P29 12. “Creo que estoy atado a mi familia más que antes.” P14 12. “I think I'm more tied to my family than I was before.” P14
	Source of meaning: love	13. “Lo más que tú quieres en la vida tus hijos, tu esposa, tu familia, tu pasión, cual tal sea.” P03 13. “The most you want in life are your children, your wife, your family, your passion, whatever it may be.” P03 14. [referring to love] “Yo lo dije…mi esposo, mis hijos, y Dios, primeramente, Dios.” P10 14. [referring to love] “I said it… my husband, my children, and God, God first.” P10 15. “Amar la familia, mis hijos, mis nietos, mis hermanas, verdad.” P21 15. “Love family, my children, my grandchildren, my sisters, right?” P21
	Source of meaning: purpose	16. “Mi propósito es (“es” mientras exhala) luchar por mi esposo. Ayudar a aquellas personas que me necesiten que estén enfermas brindarle…brindarles mis manos.” P06 16. “My purpose is (‘is' as they exhale) to fight for my husband. Helping those who need me who are sick to give. give them my hands.” P06 17. “El propósito de mi vida es…yo no esté…tratar de seguir adelante. Este…junto a mi familia.” P12 17. “My life's purpose is…Not to be…trying to move on. This…with my family.” P12 18. [referring to purpose] “Yo primero tengo que estar para mis niñas, las quiero ver crecer, quiero que ellas pues sean alguien en la vida y estén del lado de mi esposa y del lado mío.” P25 18. “First, I must be there for my girls, I want to see them grow, I want them to be someone in life and be on my wife's side and on my side.” P25
	Source of meaning: legacy	19. “Esto nos dio la oportunidad… mis hijos están más al día con su salud y eso es meta cumplida.” P13 19. “This gave us the opportunity. my children are more up to date with their health and that is a goal achieved.” P13 20. “Seguir siendo madre y seguir, tú sabes, siendo un buen ejemplo para otras mujeres que están en esta misma posición.” P24 20. “Continue being a mother and continue, you know, being a good example for other women who are in this same position.” P24
1. Social support	Family as primary social support network: after diagnosis	21. [despues del diagnostico] “Mi esposo en ese sentido es de verdad un tremendo soporte.” P02 21. [referring after the diagnosis] “In that sense, my husband truly is a tremendous support.” P02 22. [despues del diagnostico] “Y el sentido que la unión de mi familia hacia mí, todo es mucho mejor; este como que somos más unidos. Sí eso lo he encontrado, eso es algo positivo para mí, eso sí.” P12 22. [referring after the diagnosis] “And the sense of union of my family towards me, everything is much better; It's like we're more united. Yes, I have found that, that is something positive for me, yes.” P12 23. [despues del diagnostico] “Los familiares que sabían, inmediatamente buscaron a su gente más cercana y pues comencé a recibir mensajes de amor, cartas, personas que, que sí… yo sentía agrado. Y que yo podía conversar de una manera, sin sentirmeee… como cuestionada… no se o sea como… como que podían ser apoyo.” P02 23. [after the diagnostic] “The relatives who knew, immediately looked for their closest people and then I began to receive messages of love, letters, people who, yes. I felt pleased. And that I could talk in a certain way, without feeling. like questioned. I don't know, like. like they could be supportive.” P02 24. [referring to diagnosis] “Estaba mi esposo, mi mamá, mis hermanos, todos me estaban apoyando.” P10 24. [referring to diagnosis] “My husband, my mom, my brothers were there. Everyone was supporting me.” P10
	Family as primary social support network: after treatment	25. [durante el tratamiento] “Para mí, pues te digo tengo un hijo, la música, tengo muchas cosas para hacer al nivel musical, que es lo que hago, mi familia, tengo muy buenos amigos, amistades que también me han ayudado.” P03 25. [referring to treatment] “For me, well I tell you I have a son, music. I have many things to do musically, which is what I do. My family, I have very good friends, friends who have also helped me.” P03 26. [referente a la familia en el tratamiento] “Por el hecho que la fuerza que me han dado es muy grande… amistad de verdad en la manera que te ayudan y te apoyan cuando sabes que estás enfermo.” P14 26. [referring to her family during the treatment] “Because of the fact that the strength they have given me is very great… true friendship in the way they help you and support you when you know you are sick.” P14 27. [durante el trateamiento]“Si, gracias a Dios. Una de mis hijas siempre viene…” P29 27. [referring to treatment] “Yes, Thank God. One of my daughters always comes.” P29 28. [durante el tratamiento]“Cuando tú estás en esa línea que la vida es tan frágil, te das cuenta que tan importante es a veces un simple gracias, y ser agradecido, decirle al amigo, al esposo, o a la familia, gracias por estar ahí.” P30 28. [referring to treatment] “When you are on that line that life is so fragile, sometimes you realize the importance of a simple thank you, and to be grateful. Tell your friend, husband, or family, thank you for being there.” P30 29. [referiendose a esposo durante trataiento] “Ahí ha estado conmigo, gracias a Dios, y como le digo en mi caso fue para bien en muchas cosas porque se demuestra en estas situaciones que tan fuerte es el amor en una pareja, el compromiso y la ayuda… Y en este caso él ha estado conmigo, ahí está presente a lo que puede, me ayuda, me apoya…” P30 29. [referring to husband during treatment] “He has been there with me, thank God. And as I told you, in my case it was for the best in many things because in these situations it demonstrates how strong love is in a couple, the commitment and the help. And in this case, he has been with me, he is there when he can, he helps me, he supports me…” P30
	Family conflict	30. “Antes había mejor comunicación… ahora no digo nada, sufro yo solo.” P17 30. “There was better communication before. now I don't say anything, I suffer alone.” P17 31. “Él nunca ha venido al doctor conmigo, nunca, me fue infiel, y desde allí tuvimos conflicto. Me decía loca y discutíamos, no me apoyo.” P21 31. “He has never been to the doctor with me, never, he was unfaithful to me, and from then on, we had conflict. He called me crazy, and we argued, he didn't support me.” P21
2. Family communication	Planning	32. “Entonces conversé con ella, con mi hermana, no con las niñas, (risa profunda) con mi hermana y toda esta cuadrada con ella.” P02 32. “So, I talked with her, with my sister, not with the girls, (deep laughter) with my sister and everything is square with her.” P02 33. “Entonces hable con mis hermanas, acordamos el tratamiento que va a tener con él, conversamos con él, o sea todos los temas difíciles, entre comillas, porque sería la suposición de yo no estoy…” P02 33. “So I talked with my sisters, we agreed on the treatment that they're going to have with him, we talked with him, I mean, all the difficult issues (air quotes), because the assumption would be that I am not…” P02
	Lack of communication	34. “Estoy con mis padres y con dos hermanas, una estudia, otra trabaja, y entonces la comunicación no es mucha.” P15 34. “I am with my parents and two sisters, one is studying, the other one is working, so there is not much communication.” P15 35. “A veces también es falta de comunicación. Si, justo ayer le comuniqué a mi esposa que voy a terapia y ella no lo sabía. Y no eso también ha cambiado bastante, falta de comunicación. [referring after cancer diagnosis] … Los hombres no decimos nada… Ahora en ese sentido he cambiado mucho y ya no digo nada. A veces hablando. A veces discutamos.” P17 35. “Sometimes it is also a lack of communication. Yes, just yesterday I told my wife that I'm going to therapy, and she didn't know. And that has also changed a lot, lack of communication. [referring after cancer diagnosis] … We as men don't say anything… Now in that sense I've changed a lot and I don't say anything anymore. Sometimes talking, sometimes we argue.” P17 36. “No creen que uno esté enfermo, que no creen en la magnitud que uno esté enfermo, porque él me decía a mí “tú no tienes eso, que vas a tener eso, esa enfermedad no es para ti”.” P21 36. “They don't believe that one is sick, they don't believe in the magnitude that one [could be] is sick, because he told me ‘You do not have that, you are not going to have that, that disease is not for you’.” P21
3. Include family into therapy	Emotional support to family members	37. “Lo único que me preocupa, es las niñas… velar… que no pasen un duelo.” P02 37. “The only thing that worries me is the girls. to make sure. that they don't go through mourning.” P02 38. “Alguien que te pueda (pausa) este, escuchar. Y nada, todas esas cuestiones existenciales nadie te puede dar una respuesta concreta, pero si te pueden, medio quitar el sentimiento de culpabilidad… al nivel familiar, mis errores me llevaron a tener problemas en mi matrimonio. Entonces al nivel familiar me pueden ayudar.” P03 38. “Someone who can (pause) listen to you. All those existential questions, no one can give you a concrete answer, but if they can, they can partially remove the feeling of guilt. At the family level, my mistakes led me to have problems in my marriage, so at the family level they can help me.” P03 39. “Mi hijo si estaba recibiendo terapia…pero sigue igual.” P17 39. “My son was receiving therapy…but he is still the same.” P17 40. “El apoyo para la familia. Más que todo… las personas que tienen cáncer tienen menos, tienen menos problemas mentales que los que están al lado.” P20 40. “Support for the family. More than anything… people who have cancer have fewer, have fewer mental problems than those next to them.” P20 41. “Explorar y dar espacio a la gente para que se exprese… como estás y cómo va el dolor, si hay algún problema, ha habido cambios en esta semana, y cosas así… y la parte de incluir a su familia.” P25 41. “Explore and give people space to express themselves. how you are and how the pain is, if there are any problems, have there been changes this week, and things like that. and the part of including your family.” P25
	Communicate about dying	42. [referring to psychological support] “Algún momento, pues, la cuestión familiar, miedo a morirme, me da miedo y hablar de eso.” P03 42. [referring to psychological support] “At some point, well, the family issue, fear of falling asleep, it scares me and talking about it.” P03 43. “También, o sea, pensando más en ellos, en este, en ese sentido. Y, o sea, hablar de la muerte no tanto por el, el, el, el fin”. P13 43. “Also, I mean, thinking more about them in that sense. And I mean, talking about death not so much as the end.” P13

#### Theme 1: Family support

After their cancer diagnosis, participants commented about family as a source of meaning. Many participants discussed the importance of their family as a primary social network and instrumental in coping with a cancer diagnosis. The first and most frequent theme that emerged from the data was the support received by the family members and their importance in the cancer diagnosis process. Patients reported that family gives them meaning, which helps them “continue fighting.” They described how they perceived the support through gestures, conversations, letters, and kindness. The actions and attention of the family to the cancer diagnosis provided the participants meaning and support.

I believe that the only thing that has given my life meaning like this is God and my children, to continue fighting. P10

[after the diagnosis] The relatives who knew immediately looked for their closest people, and then I began to receive messages of love, letters, people who, yes… I felt pleased. And that I could talk in a certain way, without feeling… like questioned… I don't know, like… like they could be supportive. P02

#### Theme 2: Family communication

Family communication is central to “processing” the information, planning, and decision making. However, some participants noted challenges in communicating with family members due to other family responsibilities, fear of making family members suffer, and lack understanding the diagnosis. For example, a female patient reported that sometimes her sisters' and parents' responsibilities did not allow her to communicate how she felt about cancer and that she would like to discuss it. Also, a male participant reported that he prefers to “not say anything anymore” and refers to it as a cultural norm that states that men “do not say anything.”

I am living with my parents and two sisters; one is studying and the other one is working, so there is not much communication. However, I feel like I need to communicate how I felt about my diagnosis and process thing with them. P15

Sometimes it is also a lack of communication. Yes, just yesterday I told my wife that I'm going to therapy, and she did not know. And that has also changed a lot, lack of communication. [after a cancer diagnosis] … We as men don't say anything… Now in that sense, I have changed a lot, and I do not say anything anymore… Sometimes talking, sometimes we argue. P17

#### Theme 3: Include support for the family

Participants described their desire to help family members receive support and commented on their interest in encouraging family members to seek counseling. Specifically, participants would like for the family to receive services to cope with grief, find meaning, unfinished business, communicate about dying, and the emotional process.

The only thing that worries me is the girls… to make sure… that they don't go through mourning. P02

Support for the family. More than anything… people who have cancer have fewer… have fewer mental problems than those next to them. P20

Support for family members after a cancer diagnosis may help patients cope with the advanced cancer diagnosis. The inclusion of family members into therapy in the end-of-life process may help patients and caregivers with fear and emotional needs.

## Discussion and Conclusions

Emerging themes from the study include family support as a coping mechanism, family communication among Latinx individuals coping with advanced cancer, and inclusion of family support. Family as a source of meaning is a known coping skill among Latinx individuals dealing with end of life.^16,22^ In addition, it is a coping skill that often enables psychological well-being in the dyad^33^ and Latinx coping with cancer.^16^ Our findings highlight that family is a source of meaning and social support to cope with, and it produces a cancer-derived sense of purpose from their family relationships, which helped them cope with their diagnosis.

Findings also indicate the importance of improving social networks and promoting family support among patients and family members. Specifically, Latinx individuals' strong emotional bonds promote family support.^34,35^ Prior research suggests that it is essential to encourage family support by including the family in cancer care and psychosocial cancer care.^35^ Literature suggests benefiting the patients and their families by managing family-related needs and psychosocial symptoms.^36^

Participants reported their likelihood of including support for family members. In addition, patients described the importance of counseling and included the need to incorporate family into therapy after an advanced cancer diagnosis. These findings are similar to those presented by Kershaw et al^37^ and Friðriksdóttir et al,^38^ suggesting the interdependence of symptoms and the importance of simultaneous care. Specifically, these studies stated that patients' and caregivers' mental and physical health significantly influenced each other,^39^ and that family members of cancer patients who experienced symptoms of anxiety and depression reported worse quality of life than those who reported no such symptoms.^36^

In conclusion, these findings support the need to include family members and address family support when caring for Latinx patients coping with advanced cancer. Specifically, providers and interventions should include the cultural value of family relationships and family as a source of meaning in the provision of end-of-life services. Presenting Latinx cultural values in practice is essential to provide sensitive interventions among Latinx patients coping with cancer. Future research studies should address developing, adapting, and tailoring interventions for Latinx patients and families dealing with advanced cancer.

### Limitations

This qualitative study was limited, as the data are not generalizable. Also, the larger study's main goal was not the exploration of family in the context of cancer or the meaning process, and this response was derived and not further explored in the current research.

## Data Statement

The datasets generated and/or analyzed during this study are not publicly available due to Ponce Health Science University and MSK's Data Sharing Policy but are available from the corresponding author on reasonable request.

## Abbreviation Used

SD: standard deviation
